# Transverse debridement and acute shortening followed by distraction histogenesis in the treatment of open tibial fractures with bone and soft tissue loss

**DOI:** 10.1007/s11751-018-0316-z

**Published:** 2018-09-11

**Authors:** Saif Salih, Edward Mills, Jonathan McGregor-Riley, Mick Dennison, Simon Royston

**Affiliations:** Trauma and Limb Reconstruction Unit, Department of Trauma and Orthopaedics, Northern General Hospital, Herries Road, Sheffield, S5 7AU UK

**Keywords:** Open fractures, Tibia, Circular frame, Ilizarov frame, Soft tissue flap, Limb reconstruction, Distraction histogenesis, Deformity correction

## Abstract

This retrospective case series evaluates the technique of transverse debridement, acute shortening and subsequent distraction histogenesis in the management of open tibial fractures with bone and soft tissue loss, thereby avoiding the need for a soft tissue flap to cover the wound. Thirty-one patients with Gustilo grade III open tibial fractures between 2001 and 2011 were initially managed with transverse wound extensions, debridement and shortening to provide bony apposition and allowing primary wound closure without tension, or coverage with mobilization of soft tissue and split skin graft. Temporary monolateral external fixation was used to allow soft tissues resuscitation, followed by Ilizarov frame for definitive fracture stabilization. Leg length discrepancy was corrected by corticotomy and distraction histogenesis. Union was evaluated radiologically and clinically. Patients’ mean age was 37.3 years (18.3–59.3). Mean bone defect was 3.2 cm (1–8 cm). Mean time to union was 40.1 weeks (12.6–80.7 weeks), and median frame index was 75 days/cm. Median lengthening index (time in frame after corticotomy for lengthening) was 63 days/cm. Mean clinic follow-up was 79 weeks (23–174). Six patients had a total of seven complications. Four patients re-fractured after frame removal, one of whom required a second frame. Two patients required a second frame for correction of residual deformity, and one patient developed a stiff non-union which united following a second frame. There were no cases of deep infection. Acute shortening followed by distraction histogenesis is a safe method for the acute treatment of open tibial fractures with bone and soft tissue loss. This method also avoids the cost, logistical issues and morbidity associated with the use of local or free-tissue transfer flaps and has a low rate of serious complications despite the injury severity.

## Introduction

Open lower limb fractures are a common, limb-threatening presentation in adult trauma. The incidence of open tibial fractures is eight per 100,000. They are the most common open long bone fracture in adults [1]. The tibia is a subcutaneous bone along its anteromedial border, and therefore, soft tissue reconstruction is often required to treat these injuries. As well as the potential for malunion, non-union and deep infection, the soft tissue overlying the bone is susceptible to complications that may compromise limb viability.

In 2009, the British Orthopaedic Association (BOA) and British Association of Plastic and Reconstructive Surgeons (BAPRAS) updated guidelines on the management of open tibial injuries [2]. These advocate comprehensive debridement with longitudinal extension of debridement wounds along fasciotomy incisions with senior orthopaedic and plastic surgery input at the time of debridement in a specialist centre. This is to ensure adequate soft tissue coverage can be obtained. In particular, longitudinal debridement preserves the perforating arteries in the distal tibia which may form the basis for soft tissue reconstruction in this area. Immediate bony stabilization should then be closely followed by soft tissue coverage with a local or free flap within 72 h.

Flap coverage has associated risks including superficial and deep infection, donor site morbidity and a failure rate of 5–9% [3–6].

The limb salvage procedure whereby a limb is acutely shortened or deformed to facilitate soft tissue closure has been previously reported [7–9]. The technique of distraction histogenesis in trauma to restore limb length is also described [7–12]. To our knowledge, the technique of a transverse elliptical incision which closes when the limb is shortened has not previously been described. Transverse debridement utilizing an incision crossing the anterior border of the tibia against both the 2009 [2] and current [13] BOA/BAPRAS guidance is also noted.

We present a case series of 31 patients with open tibial fractures which were treated with an acute bony debridement and shortening with a transverse soft tissue debridement that enabled tension-free soft tissue coverage, and either primary closure or split skin grafting. Fracture stabilization was usually obtained with a monolateral external fixator and converted to a circular Ilizarov frame that then allowed correction of leg length discrepancy or deformity. This case series aims to evaluate the outcome of this technique as a means of dealing with appropriately selected open tibial fractures, thus avoiding the associated complications of soft tissue flaps.

## Methods

### Retrospective case review

The project is registered with the host institution’s Clinical Effectiveness Unit, and no ethical approval was required. All open fractures treated with Ilizarov circular fixators at the senior authors’ institution are logged on a database. Between January 2001 and June 2011, there were 330 open fractures added to the database. Of those, 173 were identified as having had a bone resection. From this, the open tibial fractures were identified. (Open femoral fractures and upper limb injuries were excluded.) These operation notes were reviewed, and those specifying a *transverse debridement that facilitated soft tissue closure* were included in the analysis. Any soft tissue defect that required a fasciocutaneous, local pedicled or free flap was excluded. Any case in which the split skin graft was performed by the plastic surgical team was also excluded. Non-viable bone was identified by its colour, desiccation, absence of bleeding bone ends or bone that failed the ‘tug test’ (i.e. devoid of soft tissue attachment and could be removed easily without the use of any cutting implement). All bone that was deemed non-viable was removed.

The decision to perform a transverse debridement was undertaken after consultation with a senior plastic surgeon. Injury-related and patient-related factors were considered prior to undertaking a transverse debridement. Injury factors include a transverse wound, a wound not amenable to local flap coverage, and a high-energy injury with bone loss that would result in shortening. Patient factors would include a patient who would be unsuitable for a free flap.

**Fig. 1:**
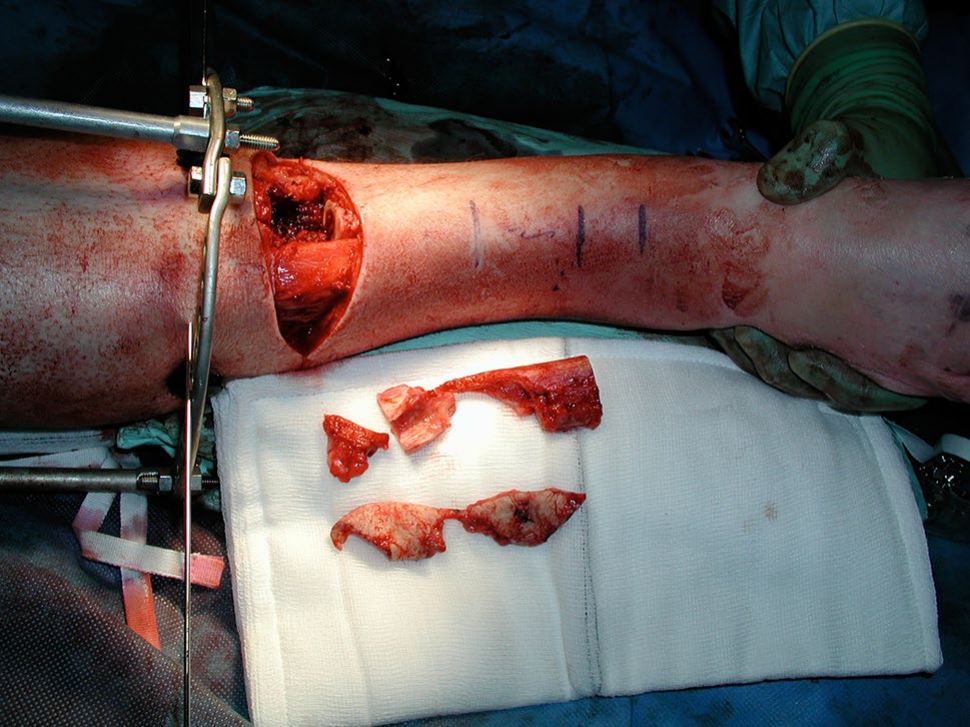
Incision made transversely across tibial crest with non-viable bone removed

**Fig. 2:**
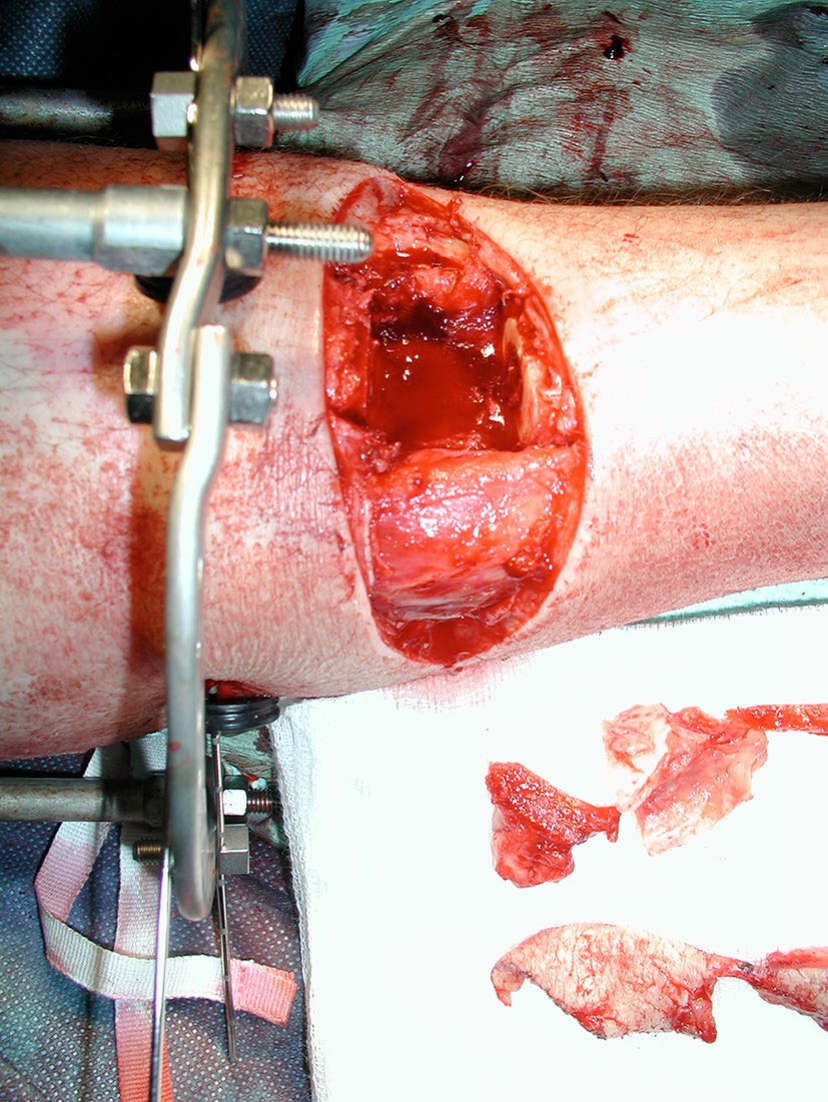
In this case, the bone ends were cut with a continuously irrigated oscillating saw to ensure sound bony apposition and a stable final construct

**Fig. 3:**
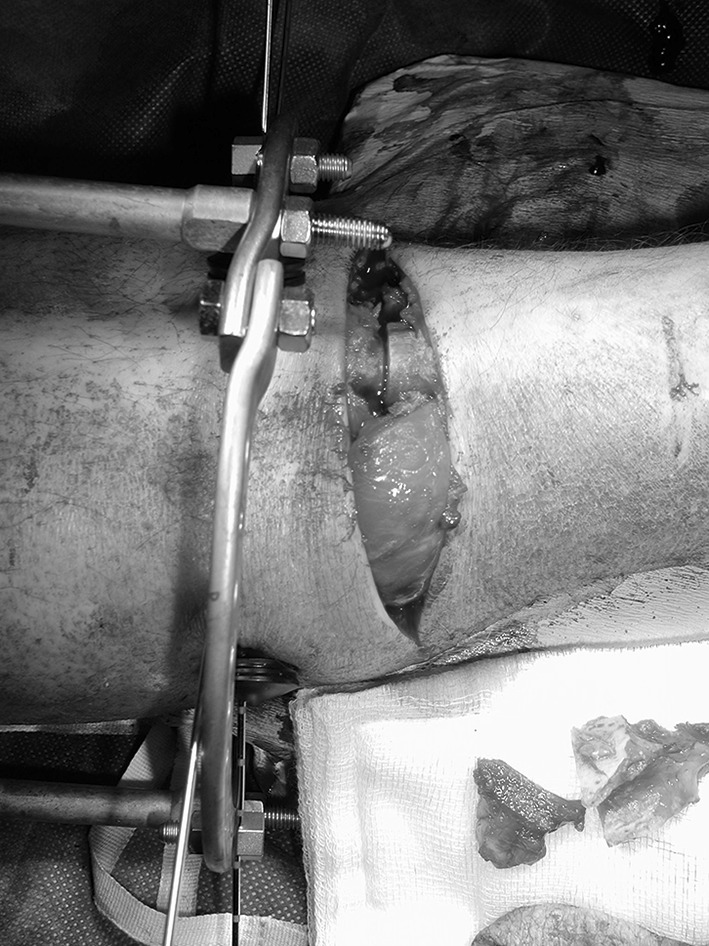
Reducing the bone ends produces the acute shortening, and the transverse elliptical incision becomes closable without tension. Final fixation was undertaken in this case with an Ilizarov circular frame, but in more recent cases a monolateral external fixator is more frequently used

Surgical debridement was performed with a tourniquet applied but not inflated unless required to control bleeding. A transverse elliptical incision across the tibial crest was made to include necrotic, grossly contaminated or unsalvageable tissue in the zone of injury and the bone ends delivered through the fracture site. Non-viable bone was removed using osteotomes (Fig. 1). In earlier cases, an oscillating saw, well cooled with saline, was used. This was used less often in the later cases in the series to minimize thermal necrosis of the bone. The bone ends were either fashioned into a peg and socket or cut flat (Fig. 2), so that there was sound apposition, depending upon the fracture configuration and what produced the most stable final construct. After bony shortening, the elliptical transverse incision could often be closed primarily (Fig. 3) and, if not, the detensioned local soft tissues were mobilized utilizing tibialis anterior, gastrocnemius or soleus to cover any exposed bone with application of a split skin graft. Usually, a temporary external fixator was used to achieve bony stability, and this was converted to a circular frame when the soft tissue envelope had healed.

**Fig. 4:**
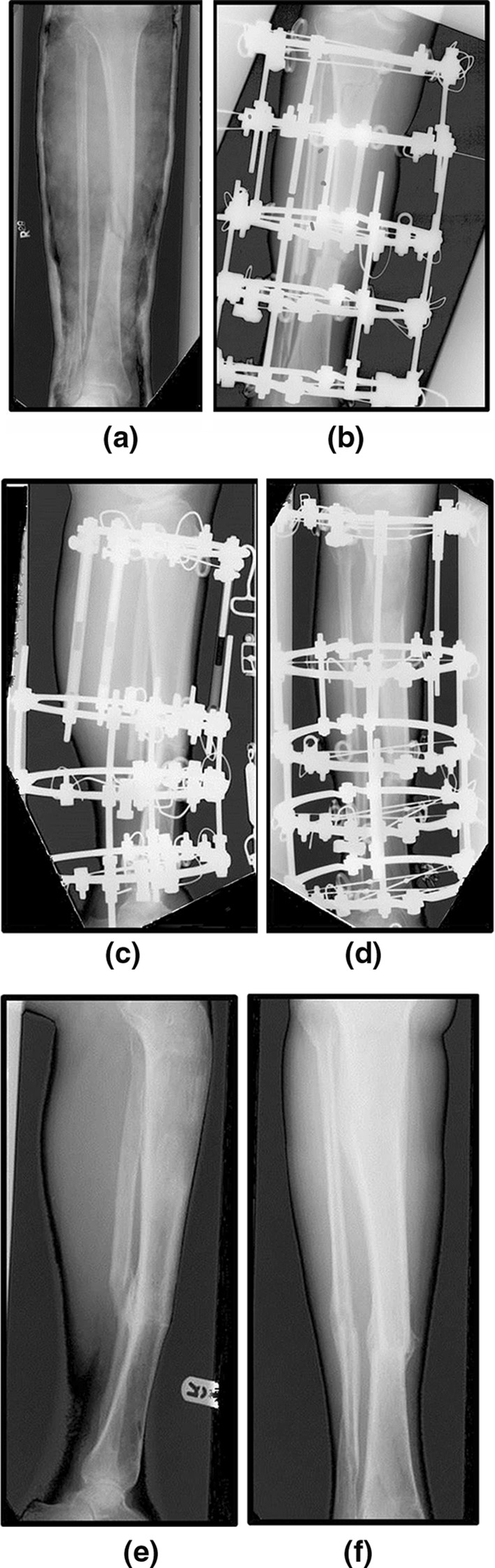
Pre-operative AP radiograph (**a**). First post-operative radiograph (**b**) showing proximal corticotomy and frame. AP (**c**) and lateral (**d**) radiographs of frame during distraction histogenesis. Final AP (**e**) and lateral (**f**) radiographs following frame removal

All but two of the patients underwent distraction histogenesis to correct leg length discrepancy. A proximal tibial metaphyseal corticotomy was performed using an osteotome. Completion was confirmed clinically and radiologically with image intensifier or intraoperative X-ray. A preoperative radiograph is shown in Fig. 4a, with this undergoing distraction histogenesis shown in Fig. 4c, d. The final radiograph post-frame removal is shown (Fig. 4e, f).

Data were then collected using a proforma, and information regarding patient demographics, comorbidities, mechanism of injury, concurrent injuries, time of initial debridement at the local receiving unit, debridement at the senior authors’ institution, date of frame application and removal, further procedures, problems, obstacles and complications were recorded. Problems, obstacles and complications were determined as previously described [14].

## Results

Thirty-one cases of open tibial fractures were identified as having undergone a transverse soft tissue debridement and a bony resection. These are summarized in Table 1. Of these, nine were in the middle third (AO classification 42) and 22 in the distal third (AO 43), of which six involved the tibiotalar joint (AO 43-B or 43-C).

**Table 1:** Data for individual patients

ID	Comorbidity	Age at time of injury	AO fracture classification	Bone loss (mm)	Closure type	Time from injury to debridement at host institution (days)	Time frame on (weeks)	Time of corticotomy post-frame (days)	Time post-fracture follow-up (weeks)	Problems, obstacles and complications
1		23.6	43-A3	20	SSG	2	22.0	0	30	
2		52.0	43-C2	20	SSG	3	14.1	0	54	
3		31.2	43-A1	20	SSG	0	25.3	0	68	2nd frame for deformity correction
4	Drug user	18.4	43-A1	50	P	3	51.7	102	62	
5	DMII, HTN, Ca breast	29.8	43-A3	25	P	4	29.1	0	174	Stiff non-union
6		51.5	43-A3	15	SSG	0	24.3	0	34	RF (PoP)
7		33.4	43-A3	20	SSG	0	23.3	0	122	FC
8	DMI, HTN	59.2	42-B1	40	SSG	4	28.4	0	65	
9		36.5	43-C3	20	P	2	43.1	112	76	
10		20.5	42-B3	30	P	2	27.9	40	41	
11	Asthma	45.7	43-C2	15	SSG	3	51.1		60	PS (overdrilling) FC
12		28.5	42-B3	10	P	1	31.1	0	42	
13		26.1	43-C3	15	SSG	3	19.3	0	74	FC
14		55.9	43-C3	25	SSG	2	27.9	0	54	
15		31.2	42-B3	30	SSG	3	43.7	0	49	
16		20.5	43-A3	25	SSG	3	25.1		88	PS
17		28.0	43-A3	80	SSG	5	58.1	0	128	
18		35.3	43-C3	40	SSG	3	50.4	71	151	PS
19		58.6	42-B2	50	SSG	57	21.3	0	67	
20		25.9	42-B2	25	P	0	54.3	62	81	PS, RF (2nd frame)
21		35.1	42-B2	30	P	10	49.3	0	139	Ring reapplication, RF (PoP)
22		41.4	43-C2	20	SSG	2	15.0	0	23	
23		41.8	43-A3	60	P	0	60.7	51	139	
24		55.8	43-C2	60	P	3	53.4	0	136	PS
25		20.1	42-A3	25	SSG	0	12.6	0	52	PS, RF (PoP), 2nd frame for deformity
26	IVDU, Etoh xs	29.9	43-A3	30	SSG	3	34.1	14	41	
27		19.4	42-B3	40	SSG	0	34.0	21	55	PS (IV)
28		34.5	43-A2	50	SSG	1	51.3	42	65	
29		51.2	42-B2	30	P	0	65.4	375	100	PS
30	RA, PE (warfarin)	57.3	42-C2	50	P	42	43.9	74	40	
31		59.3	43-A3	20	P	12	16.3	0	49	

Comorbidity: DMI, type I diabetes mellitus; DMII, type II diabetes mellitus; HTN, hypertension; IVDU, intravenous drug user; RA, rheumatoid arthritis; PE, previous pulmonary embolism. Closure type: SSG, split skin graft over soft tissue bed; P, primary closure. Problems, obstacles and complications: FC, further corticotomy; PS, pin site infection treated with oral antibiotics; OD, overdrilling of pin site to treat infection; IV, intravenous antibiotic to treat pin site infection; RF, re-fracture; PoP, treated non-operatively

Twenty-seven were male, and the median age at the time of injury was 37.3 (range 18.4–59.3). Thirteen fell from height, of which half were from ladders. Twelve were road traffic accidents, of which six were motorcyclists. Four were crush injuries and the other two were sporting injuries. The majority of patients identified were young fit males as would be expected from the high-energy mechanism that resulted in these injuries. Six patients, however, had significant comorbidities. Two were intravenous drug users, two were diabetic (one Type I and one Type II), and one was taking corticosteroids for rheumatoid arthritis. This patient was also warfarinized for recurrent pulmonary embolism.

Eleven fractures had the initial debridement performed at the host institution within 24 h of presentation. The remainder underwent initial debridement at the local receiving unit before transfer to the host institution where the second transverse debridement took place. The median time from injury to transverse debridement was 2.5 days (range 0–57 days). At the time of debridement, mean bone loss was 31 mm (range 10–80 mm). Nine frames were applied at the time of the initial debridement. Of the remainder, the mean time from debridement in the host institution to application of a circular frame was 7 days.

Wound closure was obtained primarily in 12 cases. In 19, the closure was completed with a split skin graft over a soft tissue base. In all of the described cases, closure was obtained without the need for specialist plastic surgical intervention. All of the split skin grafts had taken with none requiring further surgery.

Twenty-nine of the patients went on to have a corticotomy and distraction histogenesis. The two patients who did not undergo distraction histogenesis had bone loss at debridement of 15 mm and 25 mm, and the leg length discrepancy was treated with an orthotic. Eighteen of the 29 corticotomies were performed at the time of application of the frame. Of the remainder, the median time to corticotomy following frame application was 57 days.

The median time to union (defined as the time from injury to the time the frame was removed) was 40.1 weeks (range 12.6–80.7 weeks). Median frame index (days in frame per cm of bone loss) was 75 days/cm (mean 97 days/cm). Lengthening index was defined as the time in the frame after corticotomy per cm of bone loss was 63 days/mm (mean 80 days/cm). Patients were followed up for a median of 79 weeks (range 23–174 weeks).

### Complications

Complications are defined as issues that required further treatment or surgery after frame removal. Six patients had a total of seven complications. There were four re-fractures. Three required non-operative treatment in cast. These injuries were sustained following a fall down stairs 3 months after frame removal, jumping from a height within a week of frame removal and a simple fall sustained 9 months after frame removal. The other required a second frame before union. This case occurred 6 months after frame removal following a fall from a height. This was the only case of re-fracture following a bone resection made with an oscillating saw. Two patients required a second frame for correction of malunion. One of these patients returned for correction of an 18° varus deformity following non-operative management of a re-fracture. This was corrected gradually with an osteotomy and a hinged circular fixator. The other malunion had a 20° varus and 18° apex anterior deformity that was corrected gradually with an osteotomy and a hexapod circular fixator. One patient had a stiff non-union that required a second frame for union 23 months following removal of the initial frame. This patient was an active intravenous drug user.

### Obstacles

Obstacles are defined as issues that required further surgery but were resolved before the frame was removed. Two patients required a second corticotomy and one patient required overdrilling to treat a pin site infection. One patient required reapplication of a ring to treat symptomatic wire site.

### Problems

Problems are defined as issues that required treatment but not surgery and were fully resolved before frame removal. Pin site infection requiring oral antibiotics occurred in eight patients, of whom one also required intravenous antibiotics.

## Discussion

We present a case series of Gustilo IIIB lower limb injuries with bone loss that underwent effective limb salvage. The tibia is a subcutaneous bone, and as such, soft tissue coverage in open injuries can be problematic. The open injuries in this series were predominantly sustained in high-energy mechanisms. As a result, there were both soft tissue and bone loss. Bone loss, although not desirable, does allow reduction in tension on the surrounding soft tissues, and therefore, primary closure may be possible. The technique of deliberate bone shortening and deformity to allow soft tissue closure has been described previously for both non-unions and open fractures [8, 9]. The use of distraction histogenesis has also been extensively described to restore leg length discrepancy following trauma [7, 10–12]. To our knowledge, this is the largest case series of acute open fractures treated with acute shortening and distraction histogenesis to restore leg length.

Nho et al. [8] describe the technique of deliberate angular deformity or shortening to allow wound closure followed by a circular frame-driven deformity correction in a case example of an open fracture. In all but one of the cases in this series, the acute shortening was as a result of the bony debridement required to deal with contamination from the injury. In the other case, after consultation with the senior plastic surgeon, a decision was made and documented to resect some healthy bone to allow a primary closure. This was done to salvage the limb as the only soft tissue coverage otherwise possible would have been a free flap. In this case, a free flap was considered to be contraindicated.

El Rosasy [9] describes a similar technique for the treatment of eleven non-unions and ten acute open fractures with a 50% active infection status. This highlights the importance of a thorough debridement. In the cases reported here, there was a move away from initial debridement and frame application to initial debridement and temporary external fixator application. This allows for easier care of the soft tissues. There was also a move away from using an oscillating saw to perform any bony resection to minimize the risk of thermal necrosis of the bone. This change in practice was based on basic science principles of low-energy corticotomy applied to bony resection rather than any perceived increase in complication rate: Five of the cases had the bony resection performed with an oscillating saw, and only one developed a complication, a re-fracture. However, there were three re-fractures and a stiff non-union in the other 26 cases in which the bony resection was performed using a low-energy method (osteotome).

In 11 of the 29 cases in the series, there was a delayed corticotomy. The corticotomy was not performed at the index circular frame application if it was felt that two insults to the limb in quick succession may compromise overall union. Furthermore, the corticotomy site is above the level at which a salvage amputation would be performed. Hence, the delay was to ensure the treating surgeon was happy that the fracture was progressing to heal. The corticotomy could then be offered to the patient if they felt they had a symptomatic leg length discrepancy and without prejudice to a salvage amputation.

In the presence of bony shortening, a longitudinal debridement as advocated by the current guidelines produces a rhomboid wound which does not close easily or indeed does not close at all, requiring flap coverage. A transverse debridement not only allows an easily closable wound but also avoids the need for a soft tissue flap and the potential for flap failure or infection. Soft tissue flap complications affect up to 20% of open fractures [4–6]. Furthermore, the highest risk of flap complications occurs in the distal parts of the lower limb [15]. There were no complications associated with the soft tissue coverage in this series. Local soft tissue coverage is preferable as not only does it reduce donor site morbidity, but in high energy, combat injury there is evidence that local flaps fare better than free flaps [16]. This is a civilian case series with no ballistic or blast injuries, and hence, the pattern and severity of the soft tissue injury are different and may account for this. More recently, the use of negative-pressure dressings as an adjunct to thorough debridement has been shown to reduce the need for free-flap coverage with a subsequent reduction in the complications associated with free-flap coverage [6]. It should be stressed that negative-pressure dressings are an adjunct and are in no way a substitute for definitive soft tissue management.

There were no soft tissue or wound healing complications in this series, and all the split skin grafts were performed by the orthopaedic surgeon. All the trauma surgeons involved in performing this technique in this series have had plastic surgical training or been trained to perform split skin grafting.

Although the majority of the patients in this series are young, fit and healthy, the technique may benefit those who are at increased risk of flap failure such as the elderly, diabetic, smokers or those with multiple medical comorbidities [15]. Acute shortening may be safer in these groups. However, it is not suitable in all cases. Even in this unit where the technique is used regularly, it was applied in just over 10% of cases (32 times) out of approximately 300 open fractures over 10 years.

BOA/BAPPRAS guidelines recommend longitudinal wound extensions for debridement along fasciotomy incisions to preserve the longitudinal running neurovascular structures and perforating arteries medially and laterally that form the basis of local flap reconstructive options in the leg. Therefore, the debridement needs to be meticulous and performed by an experienced surgeon to avoid unnecessarily damaging important structures that may jeopardize future soft tissue reconstructive procedures. The host institution is a tertiary referral centre and a major trauma centre, and as such, debridement is performed by senior orthopaedic surgeons in conjunction with plastic surgeons. Although this technique has yet to produce a complication with soft tissue coverage, it is still important to have plastic surgical support in these cases as should the technique fail, the limb will almost certainly require a free flap with its associated morbidity. Careful assessment of the radiographs, the soft tissue and the pre-existing wound are made in conjunction with a senior plastic surgeon to assess the suitability of the case for transverse debridement. If doubt exists or the anticipated soft tissue defect will be larger than the anticipated bony debridement, then a traditional longitudinal debridement is undertaken so as not to exclude the possibility of a local flap.

In selected cases, a transverse wound debridement can allow early soft tissue closure and is effective in producing fracture union without the associated complications of a soft tissue flaps.
